# Intention to Extend Working Life among Thai Registered Nurses in Ministry of Public Health: A National Survey

**DOI:** 10.1155/2019/7919404

**Published:** 2019-06-16

**Authors:** Pooncharat Sirisub, Nawarat Suwannapong, Mathuros Tipayamongkholgul, Nopporn Howteerakul, Thinakorn Noree

**Affiliations:** ^1^Department of Public Health Administration, Faculty of Public Health, Mahidol University, Bangkok, Thailand; ^2^Department of Epidemiology, Faculty of Public Health, Mahidol University, Bangkok, Thailand; ^3^International Health Policy Program, Bureau of Policy and Strategy, Ministry of Public Health, Bangkok, Thailand

## Abstract

The serious shortage of registered nurses (RNs) in Thailand has made the Thai government tentatively propose a policy to extend the working life of Ministry of Public Health nurses. This study aimed to estimate the proportion of those RNs who intend to extend their working life and analyzed the associations between general characteristics, quality of work life, and job characteristics of the RNs and their intention to work past retirement age. This cross-sectional study was conducted from October 2016 to April 2017. Self-administered questionnaires were distributed nationally to 3,629 RNs in the age group 55-59 years and working for the Ministry of Public Health (MoPH), Thailand. The response rate was 85.0% (3,092 RNs). Due to the small number of male participants (n=74), males were excluded from the study. The analysis was limited to the 3,018 participants who returned the questionnaire and met the inclusion criteria. Descriptive statistics and multiple logistic regression were used for data analysis. Of the 3,018 participants, the proportion of RNs intending to extend their working life from 60 to 65 years was 30.5%. In the Service Department, the factors significantly associated with intention to extend working life were perceived good or very good health status, no shift work, monthly income more than 50,000 THB (1,595 USD), and having moderate or good working resources (p<0.01). In the four Academic Departments, perceived good or very good health status, monthly income more than 50,000 THB, family members not against the working life extension, and moderate or good working resources were the factors affecting intention to extend working life (p<0.01). This study indicated that understanding the various factors related to the intention to extend working life among RNs could lead to the design of appropriate programs to encourage them to continue working after the current retirement age.

## 1. Introduction

Shortages in nursing professionals have become increasingly prevalent in healthcare systems worldwide [1, 2]. These shortages are due to the rising demands for registered nurses (RNs), while the existing workforce ages and retires [1–3]. A number of contributing factors negatively affect this issue, including migration, threats of violence, illness, mortality, technical improvement, and retirement [4–7]. Although the global nursing professional shortage will reportedly be reduced from the current 9 million to 7.6 million by 2030, this situation will dominate global healthcare over the next 20 to 30 years [4, 5, 8]. These circumstances could precipitate direct adverse effects on healthcare systems worldwide and the well-being of people [4]. The implications of previous studies indicate that the shortage would be more serious and the supply of nursing workforce would also be inadequate to fulfill society's needs [2, 9].

Aging is the primary cause of the nursing shortage, and a number of reports have focused on this subject in various countries [7, 8]. The average age of the health workforce is on the rise, so addressing the retirement issue is important. In Thailand, the most recent available information revealed that, among 97,942 RNs, 63.4% were aged 30 to 44 years. The average age of the nursing workforce increased from 30.5 years in 2000 to 37.8 years in 2005 [10]. Data from the Thailand Nursing and Midwifery Council (TNC) from 2010 to 2016 indicated that the country needs 163,500-170,000 RNs, calculated by a nurse-to-population ratio of 1:400 [11, 12]. However, in 2010 only 130,388 RNs provided healthcare services in Thai health facilities. Therefore, during that time both the public and private sectors faced a shortage of 33,112 nurses [12]. According to the Ministry of Public Health (MoPH) [13] and the Comptroller General's Department [14], retiring Ministry of Public Health (MoPH) senior staff increased from 803 in 2011 to 2,274 in 2015; 17.6% were RNs. The number of RNs retiring increased approximately 87.8% from 2011 (213) to 2015 (400). The number of MoPH nurses retiring is likely to increase to 1,386 in 2020 [13]. This anticipated number of retiring nurses will be 4.5% of the entire anticipated health workforce. The number of nurse pensioners is predicted to be 19,490 in 2022 [10]. Although the growth rate in retirements has increased approximately 20.5% annually, 3,000-4,000 nurses are projected to retire in 2020 [10, 15]. Furthermore, in an attempt to solve the healthcare workforce shortage and lean pension budgets, the Office of the Civil Service Commission (OCSC) is determined to extend the retirement age from 60 to 65 due to the increased average age of the health workforce in Thailand [16].

Another common factor contributing to the nursing shortage is the high turnover rate [11, 12]. Many RNs retire early due to poor work conditions, such as heavy workloads, low job control, and inadequate social support in the workplace [11, 15]. Other findings in Thailand revealed the exit rate of nurses of 4.4% annually and this percentage is expected to increase to over 15.0% by 2020 [10]. A study reported that 30.0% of hospital nurses in Thailand are inclined to leave their profession [11]. The consequences of previous circumstances are likely to result in a human resource deficit [10–14]. In 2014, the records of the TNC showed a massive loss of 5,800 nurses, generally due to retirement, early retirement, and job changes. In 2009 the MOPH revealed that a deficit of 40,000 personnel remains, even though the MoPH planned to produce 10,000 RNs a year [10]. A 20-year longitudinal study of the professional lives and health of nurses (2009-2027) indicated that new RNs are willing to continue working at rates of 99, 98, 95, 80, and 80 in the first, second, third, fourth, and fifth years, respectively [7]. Because evidence suggests that the reduction of RNs could produce detrimental effects on the outcomes of patients, concentrating on this matter is essential to alleviate the impact of the consequences [11, 15].

Thailand is becoming an aging society, and the country's economic situation is predicted to be negatively affected over the next 10-15 years [10, 11] as the retirement age for all civil servants is 60 years old. Extending the working life is an alternative to delaying pension payments for government workers [16]. The extension would reduce budget deficits in the future [16, 17]. A large number of retired, healthy people who are full of relevant experience, professional skills, and knowledgeable expertise might be available to continue working. As they could pass on their experiences to the next generation and make great contributions to society, it would be favorable to let them remain employed [16–18]. However, delaying retirement might prevent the younger, newly graduated generation from being employed, while also potentially hindering the promotion of existing staff into recently vacated positions. The working extension cannot be implemented immediately as it requires a large budget and limited position available. The Thai government has started its strategy by extending according to the need of the department. Nonetheless, the development and implementation of effective strategies supporting RNs retention beyond the usual retirement age require a clear understanding of the significant factors affecting intention to remain in the nursing profession [17].

Deciding when to retire may be one of the most important decisions an individual can make during his or her lifetime. Although the retirement decision occurs late in life, it can dramatically affect the well-being of a person for many years [3]. Many factors determining whether older professionals are able to extend their ability to work are complex and involve many different perspectives and research areas [3, 4, 6, 19, 20]. Health is an essential factor correlating to the ability of older professionals to participate in work life and a key determinant of the extension of a working life [3, 4, 21, 22]. The intention to extend working life is not always entirely voluntary as it may be affected by the individual's financial situation [19, 20, 23]. Having financial incentives is beneficial for the health of older people when they choose to continue their career [20, 23].

Quality of work life (QoWL) is another important criterion requiring focus by organizations to achieve higher productivity and organization goals, while also retaining the current workforce [24–26]. The term “quality of work life” was originated by Davis [27] in late 1960s but its measurable dimensions were delineated by Walton [28] in 1975. There have been many studies in various groups but, among health professionals, nurse quality of work life (NQWL) has been the subject of most investigations. Knox and Irving [24] presented 14 factors comprising NQWL. They are the following: reduced work stress, organizational commitment and belonging, positive communication with supervisors, autonomy, recognition, reutilization, predictability of work activities, fairness, clear locus of control of organizational decisions, education, professionalism, low role conflict, job performance feedback, opportunity for advancement, and fair and equitable pay levels. These 14 factors were concluded into 8 dimensions: organizational structure and function, individual staff perceptions, scope and complexity of role, career paths, collaborative communication, working environment, nature of work, and working resources.

Job characteristics and work satisfaction have been found to be significant factors in determining decisions to retire or extend working life [29–31]. In 1974 Hackman and Oldham [32] introduced the Job Characteristics Model (JCM) which has five core job dimensions identified as skill variety, task identity, task significance, autonomy, and job feedback. They defined skill variety as the degree to which a job requires a variety of different activities in carrying out the work, which involve the use of a number of skills and talents of the employee. Coelho and Augusto [33] stated that task identity encourages the feeling that job is meaningful and worthwhile thus motivating the employee to work effectively. Hackman and Oldham [32] defined task significance as the degree to which a job has a substantial impact on the lives or work of other people. They also explained that autonomy is the degree to which a job provides freedom, independence, and discretion to the employees in scheduling his or her work and in determining the procedures to be used in carrying it out.

The MoPH continues to face nursing shortages and little information is available on RNs extending their working life. Therefore, this study aimed to estimate the proportion of RNs who intend to extend their working life and the associations between their general characteristics, QoWL, job characteristics, and intention to extend working life. The findings could be of much benefit to MoPH policymakers and those involved in health personnel planning.

## 2. Methods and Materials

### 2.1. Study Sites and Study Samples

Between October 2016 and April 2017, a self-administered questionnaire was sent to 3,629 RNs near retirement age (i.e., those aged 55 to 59 years) working nationally for the MoPH, Thailand. The 3,629 were randomly selected from the 108,348 RNs near retirement across the five departments of the MoPH. The sample size allowed for a low response rate and the risk of posted questionnaires being lost.

Due to the differences in organization and working contexts, this study categorized the study sites into two types, Service Department (SD) and Academic Departments (AD), of which there were four. The mission of the SD is focused on nursing services, disease prevention, health promotion, and rehabilitation. On the other hand, the four AD provide technical support on care provision, disease prevention, health promotion, and rehabilitation to the SD.

The inclusion criteria were RNs, aged 55-59 years, working with MoPH, and willing to participate in the study. RNs suffering from serious illness and cognitive impairment, those who had already early retired, and those who had transferred to another job were excluded. RNs who met the criteria of the study in the SD was 3,214 and, for the four AD, the number was 415. The response rate was 85% (3,092 RNs). Due to the small number of male participants (n=74) we limited the analysis to only female RNs, which made a total number of 3,018 RNs. 2,622 RNs were from the SD and 396 were from AD.

### 2.2. Measures

Self-administered questionnaires were used in this study. Three experts from the TNC assessed and offered suggestions on the content validity of the questionnaire. The revised version was pretested with 30 RNs affiliated to the Bangkok Metropolitan Administration and the questionnaires were revised again before being distributed to the sample population.

General characteristics included age, educational level, marital status, family members against working life extension or not, years of working, shift work, monthly income, and perceived health status.

Quality of work life (QoWL) was measured using the concept formulated by Knox and Irving [24] that fit to the transformation period of healthcare reform in Thailand. QoWL scale had 30 items on a five-point Likert type rating scale: ranging from “strongly disagree” = 1 to “strongly agree” = 5. QoWL was measured by eight subscales: (1) organizational structure and function, with five items and a Cronbach's alpha coefficient of 0.906; (2) individual nurse perceptions, with three items and a Cronbach's alpha coefficient of 0.909; (3) scope and complexity of role, with four items and a Cronbach's alpha coefficient of 0.908; (4) career paths, with six items and a Cronbach's alpha coefficient of 0.907; (5) collaborative communication, with three items and a Cronbach's alpha coefficient of 0.907; (6) working environment, with three items and a Cronbach's alpha coefficient of 0.908; (7) nature of work, with three items and a Cronbach's alpha coefficient of 0.909; and (8) working resources, with three items and a Cronbach's alpha coefficient of 0.908.

Job characteristics were primarily based on the concept of Hackman and Oldham [32]. Job characteristics scale had 15 items on a five-point Likert's type rating scale, ranging from “strongly disagree” = 1 to “strongly agree” = 5. Job characteristics had five subscales: (1) skill variety, with two items and a Cronbach's alpha coefficient of 0.857; (2) task identity, with three items and a Cronbach's alpha coefficient of 0.863; (3) task significance, with three items and a Cronbach's alpha coefficient of 0.866; (4) job autonomy, with four items and a Cronbach's alpha coefficient of 0.860; (5) job feedback, with three items and a Cronbach's alpha coefficient of 0.855.

The questions asked about the proposed policy to extend working life of healthcare professionals from 60 to 65 years. One question asked RNs about their intentions regarding retirement if the government implemented the policy to extend working life. The possible answers were the following: (1) extend working period until the age of 65; (2) retire at the age of 60, and (3) uncertain. The final part of the questionnaire was an open-ended question about participants' suggestions for the MoPH on how to make RNs extend their working life. We determined the criteria for group classification of QoWL and job characteristics by group references and divided the scores into three levels: good (percentile 75), moderate (percentile 50), and poor (percentile 25).

### 2.3. Data Analysis

Data analysis was carried out using SPSS Software, Version 18, under the license of Mahidol University (IBM Corp, Armonk, NY, USA). Descriptive statistics were used to describe each variable. Frequency, percentage, median, range, and percentiles 25, 50, and 75 were used. The level of statistical significance was set at p<0.05. Multiple logistic regression analysis was applied to identify the associations between general characteristics, QoWL, job characteristics, and intention to delay retirement from 60 to 65 years. All study variables were selected in the multiple logistic regression models using the forward method.

### 2.4. Ethical Considerations

We received ethics approval from the MoPH, Thailand (Document No. 38/2016), and the Ethics Review Committee of the Faculty of Public Health, Mahidol University (COA. No. MUPH 2016-098).

## 3. Results

### 3.1. General Characteristics of Registered Nurses

About 86.9% (2,622 RNs) worked in the SD. In this group 73.5% held a Bachelor degree in Nursing Science or equivalent. 65.2% were married and 52.9% lived in an extended family. 65.7% reported that their family members were not against a working life extension. 76.9% had more than 30 years' working experience. 68.4% reported they had no shift work. Among RNs who had shift work, 60.3% reported they had shift work less than or equal to five times per month. More than three-fourths (78.9%) had monthly incomes more than 50,000 THB (1USD = 31.35 THB). 42.8% had chronic diseases, such as hypertension and musculoskeletal disorders which did not affect their work. Over two-thirds (69.9%) perceived their health status as good (data not shown).

About 13.1% of the nearly retiring RNs (396/3,018) worked in the four AD. In this group most (78.0%) held a Bachelor degree in Nursing Science or equivalent and more than half (68.9%) were married and lived in a nuclear family (57.1%). 50.8% reported that their family members were not against their plan to extend their working life. 83.8% had more than 30 years' working experience. 43.3% reported they had shift work. Among the participants who had shift work, 90.1% reported they had shift work less than or equal to five times per month. About 42.9% had a monthly income of more than 60,000 THB. 56.6% reported they had chronic diseases, such as hypertension and musculoskeletal disorders which did not affect their work (data not shown).

### 3.2. Proportions of Registered Nurses Intending to Extend Their Working Life from 60 to 65 Years

Of the 3,018 RNs, 30.5% (919) reported an intension to retire at 65 years old. The percentage varied by study site, with about 31.0% (813/2622) from the SD stating they intend to extend, with the percentage being 26.8% (106/396) in the four AD (Table 1).

### 3.3. Reasons and Nature of Work of RNs Who Intend to Delay Their Retirement to 65 Years Old

Table 2 shows that, among those who intend to extend their working life, most of them (74.1%) held a Bachelor degree, 66.4% were married, and 56.5% lived in a nuclear family and did not have family against their intention to extend their working life. It was also observed that 67.4% had no shift work and where there was shift work, it was less than or equal to 5 times a month. Among the group, 84.2% had a monthly income higher than 50,000 THB; 71.3% had a perceived health status of good and very good. The three top reasons participants expressed for their intention to extend their working life until 65 years were the following: (1) healthy and able to work (96.2%); (2) having knowledge, potential, expertise, and experience in the nursing profession (90.2%); and (3) they could earn income after retirement (86.6%). One third of them (37.1%) said they would like to get a new job and new position corresponding to their knowledge and skill and 30.6% would like to work in the same job and same position until 65 years, as shown in Table 3. The situation was similar between the SD and the AD.

### 3.4. Quality of Work Life and Job Characteristics

Most participants (60.0%) in the SD had an overall moderate QoWL. Good QoWL was found in collaborative communication (24.7%), work environment (24.4%), and individual nurse perceptions (23.9%). Collaborative communication is defined as the ability of a nurse to exchange and provide information during communication with colleagues and patients. Work environment refers to an environment that encourages RNs to use medical equipment effectively during the course of change in addition to participating in adjustment for an optimal working environment. Individual nurse perceptions refer to recognizing the value of nurse's profession and the individuals' enthusiasms in each circumstance such as function and responsibility, giving new idea, and recognition that could promote and sustain QoWL [24]. The majority of the participants (62.6%) in the four AD had an overall moderate QoWL. Almost half of the AD participants (45.5%) reported their career paths were at the poor level as shown in Table 4.

Approximately half the participants (58.2%) in the SD reported a moderate level of overall job characteristics. 59.3% reported they had a good level of skill variety. 23.0% had poor level of task identity. About 46.2% of participants in AD had an overall moderate level of job characteristics. 41.9% reported that they had a good level of skill variety, whereas 38.4% had poor level of job feedback as shown in Table 4. Skill variety refers to the need to use a number of different skills and personal talents to carry out the work. Task identity is defined as the degree of requirements of RNs in the completion of a whole, identifiable piece of work from the beginning to the end with observable outcomes. Job feedback refers to how much direct and clear information about the effectiveness of their performance individuals receive [32].

### 3.5. Factors Associated with Intention to Extend Working Life

In the multivariate analysis, all significant variables in the univariate analyses were simultaneously entered into the two separated final logistic regression models to identify, using the forward method, the significant predictors of intention to extend working life.

In the SD, the factors significantly associated with intention to extend working life were the following: perceived health status at good or very good levels (aOR=1.58, 95% CI=1.22-2.04), no shift work (aOR=1.78, 95%CI= 1.33-2.38), monthly income equal to or more than 50,000 THB (aOR= 1.42, 95% CI=1.13-1.80), and working resources at moderate or good levels (aOR=2.53, 95% CI= 1.80-3.58).

In the AD, the factors significantly associated with intention to extend working life were the following: perceived health status at good or very good levels (aOR=2.27, 95% CI=1.26-4.12), monthly income equal to or more than 50,000 THB (aOR= 2.48, 95% CI=1.38-4.46), family members not against extension (aOR=2.15, 95% CI=1.27-3.64), and moderate or good working resources (aOR=3.74, 95% CI=1.82-7.66) (Table 5).

## 4. Discussion

The main finding of this study indicated that 30.5% of RNs in the MoPH intended to extend their working life to 65 years old. This proportion can be used to predict the number of RNs in the future as it is a national survey. The finding was similar to a Korean study (32.8%) [34] in which the limited availability of alternative jobs was acknowledged with perceived augmented costs potentially leading to RNs developing extension commitment. The Korean study collected data from the nurses of all age groups but the results were similar to our study that was conducted in RNs near retirement age. However, the percentage of the current study was lower than the findings of studies in the United States (83.8%) [35] and Taiwan (50.8%) [36], where RNs have higher salaries and more incentives. These different findings might be due to the complexity of intention for working life extension. Previous studies have found that the factors affecting the intention of individual RNs vary based on personal characteristics, quality of nursing work life, and years of working [3, 19, 21, 22, 30, 31, 37].

### 4.1. Service Department

In the SD, the proportion of RNs intending to extend their working life was 31.0%. Although 41.3% had shift work, they considered that their work was an important part of their lives and perceived that they were healthy and able to work (96.2%). Personal economic incentives or the need to earn income after retirement was reported by 86.6%. About one-third intended to extend their working life if they were offered a new job or position commensurate with their knowledge and skills (Table 2). This implied that they wanted to reduce their workload, be reassigned to less physically demanding jobs such as administrative work or being an instructor, and access flexible work arrangements, which might take the form of shorter shifts, casual hours, or part-time work [4, 19, 38]. About 31.3% had an educational level of Master's degree or higher. Most (88.9%) said that RNs had knowledge and more experience in the nursing profession and approximately 81.9% said they could contribute to the betterment of society. This indicated that they felt recognized and respected and that their knowledge, experience, and perspectives were appreciated [39, 40]. In addition, the RNs valued the ability to continue learning and developing themselves professionally. They also expressed there was a need to redesign jobs for older nurses and offer job rotation opportunities.

RNs with a perceived health status of good or very good were 1.58 times more likely to extend their working life than those who had moderate health status (aOR=1.58, 95% CI=1.22-2.04). The reasons might be they perceived that they had good health status and were able to work. However, 37.5% said they wanted to work in new jobs or positions commensurate with their knowledge and skills after extending. The findings of the current study agreed with those from similar studies [21, 22, 37, 41]. Many related studies [24, 25, 42, 43] found self-perceived health status was important in explaining the self-perceived ability to continue working until or beyond retirement age.

RNs who had no shift work were 1.78 times more likely to extend their working life than those who had shift work (aOR=1.78, 95%CI=1.33-2.38). Shift work is traditionally a significant source of stress among RNs. In this study, the RNs who intended to extend their working life reported that they were looking for job flexibility and less stressful work after retirement. This finding matches those of similar studies [20, 38, 40, 44–46]. Shift work is highly unwanted by nurses as they grow older and nurses who continue to perform shift work are less likely to work until they are 65 years of age. RNs who earned more than 50,000 THB per month were 1.42 times more likely to extend their working life compared to those earning less (aOR=1.42, 95%CI=1.13-1.80). Some participants stated that they might continue to work if they had not saved enough money for retirement or need to continue to support dependents financially. This finding is similar to previous studies that older nurses were more likely to work until 65 years old and beyond when they or their family members had not saved enough to achieve their retirement plans or were financially unprepared for retirement [19, 23, 47]. The findings from these related studies revealed that older nurses continue to work to positively affect their financial situation in the future, or to finance the postretirement lifestyle they desired. They were motivated to not retire by financial incentives and ideal hourly rates.

RNs who had working resources at moderate and good levels were 2.53 times more likely to extend working life than those with poor working resources (aOR=2.53, 95%CI=1.80-3.58). Other studies have shown that RNs frequently perceive a higher QoWL when working resources are available, and there is an associated high level of accountability for the productivity and outcomes associated with these working resources. This study had similar findings to other studies [24, 26, 48] that found that human, financial, and material resources are critical issues when redesigning of resources allocation in an organization. In order to retain older RNs in the MoPH, the availability of working resources that will assist in maintaining the QoWL for nurses should be addressed. These include education, rewards, and recognition for performance and administration support [49, 50].

### 4.2. Academic Departments

In the four AD, the proportion of RNs who intend to extend their working life was 26.8%. All of those who intend to extend perceived that they possess knowledge and experience in the nursing profession, and approximately 96.2% stated that they would be able to work until 65 years old due to their good health status. All of them (100%) stated their need to be respected, accepted, and credited for their expertise [4, 20, 23, 48].

RNs who perceived their health status as good and very good were 2.27 times more likely to extend their working life than those who perceived moderate health status (aOR=2.27, 95%CI=1.26-4.12). The reasons might be they perceived that they had good health status and were able to work. However, 34.0% RNs wanted to work in new jobs or positions commensurate with their knowledge and skills after retirement, the same as RNs in the SD. The findings of the current study are congruent with those of similar studies [20, 21, 37, 41].

The study showed that RNs who had monthly incomes exceeding 50,000 THB were 2.48 times more likely to extend their working life than those who earned less than or equal to 50,000 THB (aOR=2.48, 95%CI=1.38-4.46). This might be because they were satisfied with their earnings and they valued being financially recognized including their expertise and work performance. This matches the findings of previous studies [19, 20, 40, 47]. Financial consideration was a key factor that encouraged nurses to extend their working life.

RNs who had family members not against their intention were 2.15 times more likely to intend to continue working life than those whose family members were against their intention (aOR=2.15, 95%CI= 1.27-3.64). This might be because their family members understood their work and did not want to interfere with their work or intention about retirement. However, the findings of this study differ from other studies [19, 40, 51] that found that family factors might influence the retirement process, either through involvement in or influence on retirement plans. RNs in our study frequently perceived that their family agreed with their intention regarding working because they had freedom in performing work, making decisions regarding work, and enjoying opportunities to balance their work schedules [37, 38, 50]. This finding is similar to those of other studies across all occupations [20, 40, 51] that found that workers aged 50 years and older found it easier to balance their working hours with family and social commitments than younger workers. If work provides opportunities for relaxation activities and meeting family commitments, retirement may be delayed [52, 53]. RNs who had working resources at moderate and good levels were 3.74 times more likely to continue working life than those who had poor working resources (aOR=3.74, 95% CI=1.82-7.66). This indicates that RNs consider continuing to work when they are sufficiently supported with manpower, financial resources, materials, and equipment [49, 50].

In this study, 96.2% of those who intend to extend their working life cited their work ability as a reason. Work ability (WA) is the ability of the worker to perform his or her tasks at work. WA is the outcome of the balance between the individual's resources and work-related aspects [42]. It is conditioned by work demands, health status, and physical and mental abilities. WA has a strong health-related nature; thus, it inevitably decreases with age, even if there is a higher rate of variability among individuals. WA is associated with a high quality of work, high productivity, and enjoyment of time at work. WA also forecasts a good quality of life, well-being, and active and meaningful retirement [54].

### 4.3. Strengths and Limitations of the Study

This was the first national survey of Thai MoPH RNs near retirement age (55-59 years). The estimated proportion of intention to extend working life can be used for projection of the number of RNs in the future. The factors affecting the intention to delay retirement should be considered by the MoPH. However, as the responses relied on self-reporting, the measurements might be limited.

## 5. Conclusion

This study had a large sample size and covered all RNs working in MoPH. The projected number of RNs using the estimated proportion from this study could be crucial information for policymakers trying to solve problems related to the RNs shortage. MoPH senior managers should consider the various factors influencing RNs' intentions to extend their working life and use active workplace interventions related to maintaining the health and well-being of RNs, setting regulations for optimal numbers and arranging preferred shift work schedules. They should provide RNs a reasonable monthly income compared to their workload that is in line with their WA and also provide benefit systems, such as bonuses or other related incentives. Senior managers should optimize nurse workloads. RNs should be provided sufficient working resources and opportunities to participate in job design and create their own working methods. Family members should be helped to have a better understanding of the task significance of the RNs so there will be more family support for working life extension. Future mixed-methods research should be carried out to gather and analyze the viewpoints of policymakers and planners concerning the proposed policy to extend the working life of MoPH RNs. Assessing WA among RNs should be conducted to determine an individual's ability to work after the usual retirement age.

## Figures and Tables

**Table 1:** Proportions of registered nurses who intend to extend their working life (n=3,018).

Department	Retirement intention						
	Total	Retire at 60 years old		Retire at 65 years old		Uncertain on retirement age	
	n	n	%	n	%	n	%
Service Department^a^	2,622	1,329	50.7	813	31.0	480	18.3

Academic Departments^b^	396	194	49.0	106	26.8	96	24.2

Total	3,018	1,523	50.5	919	30.5	576	19.1

Notes: ^a^the Service Department focuses on nursing services, disease prevention, health promotion, and rehabilitation; ^b^the Academic Departments provide technical support to the Service Department on care provision, disease prevention, health promotion, and rehabilitation.

**Table 2:** Individual characteristics of registered nurses who intend to extend their working life until 65 years old (n=919).

Characteristic	Total (n=919)		Service Department		Academic Departments	
			(n=813)		(n=106)	
	n	%	n	%	n	%
Educational level						

Bachelor degree or equivalent	681	74.1	595	73.2	86	81.1
Master degree or higher	238	25.9	218	26.8	20	18.9

Marital status						

Single	131	14.2	108	13.3	23	21.7
Widowed/Divorced/Separated	178	19.4	163	20.0	15	14.2
Married	610	66.4	542	66.7	68	64.2

Family type they live with						

Nuclear family	519	56.5	469	57.7	50	47.2
Extended family	400	43.5	344	42.3	56	52.8

Family member against extension						

No	621	67.6	554	68.1	67	63.2
Yes	298	32.4	259	31.9	39	36.8

Shift work						

No	619	67.4	571	70.2	48	45.3
Yes	300	32.6	242	29.8	58	54.7

Frequency of shift work (n=282)						

≤5 times per month	247	87.6	193	23.7	54	50.9
>5 times per month	35	12.4	32	3.9	3	2.8

Monthly income (THB)^a^						

<50,000	145	15.8	125	15.4	20	18.9
50,001-60,000	375	40.8	345	42.4	30	28.3
>60,000	399	43.4	343	42.2	56	52.8

Perceived health status						

Very good	56	6.1	53	6.5	3	2.8
Good	599	65.2	552	67.9	47	44.3
Moderate	264	28.7	208	25.6	56	52.8

Notes: ^a^1USD = 31.35 Baht.

**Table 3:** Reasons and nature of work wanted of 919 registered nurses who intend to delay their retirement until 65 years old.

	Total		Service Department		Academic Departments	
	(n=919)		(n=813)		(n=106)	
	n	%	n	%	n	%
*Reason given by registered nurses who plan to delay retirement until 65 years old* ^*a*^						

Healthy and able to work	884	96.2	782	96.2	102	96.2
Educated and have potential, expertise and experience in nursing	829	90.2	723	88.9	106	100.0
Earn income after retirement	796	86.6	700	86.1	97	91.5
Value and benefit to society	748	81.4	666	81.9	82	77.4
Reduce the professional nurse shortage	233	25.4	197	24.2	36	34.0
Other (e.g., have spouse or close friends at work and their daily life)	70	7.6	63	7.7	7	6.6

*Nature of work wanted by registered nurses who plan to delay retirement until 65 years old*						

New job or position corresponding to knowledge and skill	341	37.1	305	37.5	36	34.0
The same job and position until 65 years old	281	30.6	244	30.0	37	30.9
Same position but in part time job	155	16.9	136	16.7	19	17.9
New job and it is a part time job	142	15.5	128	15.7	14	13.2

*Notes*. ^a^Multiple responses were allowed.

**Table 4:** Registered nurses' quality of work life and their job characteristics (n=3,018).

Variables	Service Department			Academic Departments		
	(n=2,622)			(n=396)		
	Poor	Moderate	Good	Poor	Moderate	Good
*Overall quality of work life*	*15.3*	*60.0*	*24.7*	*28.0*	*62.6*	*9.3*

Organizational structure and function	13.8	63.5	22.8	19.4	65.9	14.6

Individual nurse perceptions	14.3	61.9	23.9	28.8	58.3	12.9

Scope and complexity of role	16.2	68.3	15.5	29.5	63.9	6.6

Career paths	18.1	63.3	18.6	45.5	43.7	10.9

Collaborative communication	15.3	60.0	24.7	28.0	62.6	9.3

Work environment	14.0	61.6	24.4	18.7	67.9	13.4

Nature of work	9.9	75.1	15.1	12.6	77.0	10.4

Working resources	15.6	61.9	22.5	26.5	50.5	23.0

*Overall job characteristics *	*18.2*	*58.2*	*23.6*	*39.1*	*46.2*	*14.6*

Skill variety	2.2	38.5	59.3	1.0	57.1	41.9

Task identity	23.0	56.9	20.2	36.6	50.8	12.6

Task significance	20.2	39.4	40.4	26.0	33.8	40.2

Job autonomy	14.6	71.1	14.3	7.8	58.6	33.6

Job feedback	19.0	58.4	22.7	38.4	45.7	15.9

**Table 5:** Multiple logistic regression of intention to extend working life among registered nurses (n=3,018).

Variables	Service Department (n=2,622)			Academic Departments (n=396)		
	aOR	95% CI	p-value^a^	aOR	95% CI	p-value^b^
Age (55-59 years)^a^	1.04	0.96-1.12	0.384	0.94	0.77-1.17	0.591

Perceived health status						

Moderate	Ref			Ref		

Good or very good	1.58	1.22-2.04	0.001	2.27	1.26-4.12	0.007

Shift work						

Yes	Ref			Ref		

No	1.78	1.33-2.38	<0.001	1.31	0.71-2.43	0.387

Monthly income (Baht)						

Less than or equal to 50,000	Ref			Ref		

More than 50,000	1.42	1.13-1.80	0.003	2.48	1.38-4.46	0.002

Family member against extension						

Yes	Ref					

No	1.21	0.99-1.48	0.058	2.15	1.27-3.64	0.004

Working resources						

Poor	Ref			Ref		

Moderate or good	2.53	1.80-3.58	<0.001	3.74	1.82-7.66	<0.001

*Notes*. Perceived health status refers to the registered nurse's feeling concerning the state of her health. Shift work refers to a work schedule that includes frequent periods of nonstandard work hours, compared to a fixed daily work schedule with standard day work hours. Monthly income refers to the total monthly wage or salary. It includes basic pay, overtime pay, and other allowances. Family members not against extension means the nurses can decide by themselves about their extension. Working resources refer to efficiency of manpower, financial, materials, and equipment which are used at the facility where the registered nurse works; ^a^continuous data. ^b^Significance was set at p<0.05.* Abbreviations*. aOR, adjusted odds ratio; Ref, reference group.

## Data Availability

The data used to support the findings of this study are available from the corresponding author upon request.
